# Serum procalcitonin for the early recognition of nosocomial infection in the critically ill patients: a preliminary report

**DOI:** 10.1186/1471-2334-9-49

**Published:** 2009-04-22

**Authors:** Pierre Emmanuel Charles, Emmanuel Kus, Serge AHO, Sébastien Prin, Jean-Marc Doise, Nils-Olivier Olsson, Bernard Blettery, Jean-Pierre Quenot

**Affiliations:** 1 Service de Réanimation Médicale, Hôpital Le Bocage, CHU de DIJON, France; 2 Service d'Epidémiologie et d'Hygiène Hospitalière, Hôpital Le Bocage, CHU de DIJON, France; 3 Laboratoire d'Immunologie, Hôpital Le Bocage, CHU de DIJON, France

## Abstract

**Background:**

The usefulness of procalcitonin (PCT) measurement in critically ill medical patients with suspected nosocomial infection is unclear. The aim of the study was to assess PCT value for the early diagnosis of bacterial nosocomial infection in selected critically ill patients.

**Methods:**

An observational cohort study in a 15-bed intensive care unit was performed. Seventy patients with either proven (n = 47) or clinically suspected but not confirmed (n = 23) nosocomial infection were included. Procalcitonin measurements were obtained the day when the infection was suspected (D0) and at least one time within the 3 previous days (D-3 to D0). Patients with proven infection were compared to those without. The diagnostic value of PCT on D0 was determined through the construction of the corresponding receiver operating characteristic (ROC) curve. In addition, the predictive value of PCT variations preceding the clinical suspicion of infection was assessed.

**Results:**

PCT on D0 was the best predictor of proven infection in this population of ICU patients with a clinical suspicion of infection (AUROCC = 0.80; 95% CI, 0.68–0.91). Thus, a cut-off value of 0.44 ng/mL provides sensitivity and specificity of 65.2% and 83.0%, respectively. Procalcitonin variation between D-1 and D0 was calculated in 45 patients and was also found to be predictive of nosocomial infection (AUROCC = 0.89; 95% CI, 0.79–0.98) with a 100% positive predictive value if the +0.26 ng/mL threshold value was applied. Comparable results were obtained when PCT variation between D-2 and D0, or D-3 and D0 were considered. In contrast, CRP elevation, leukocyte count and fever had a poor predictive value in our population.

**Conclusion:**

PCT monitoring could be helpful in the early diagnosis of nosocomial infection in the ICU. Both absolute values and variations should be considered and evaluated in further studies.

## Background

In critically ill patients, nosocomial infection is generally associated with an increased risk of death and a greater length of stay [1]. The most frequently encountered intensive care unit (ICU)-acquired infections are ventilator associated pneumonia (VAP) and bacteremia [2,3]. Outcome can be improved if a prompt and appropriate antibiotic therapy is administered [4]. In contrast to patients with community-acquired sepsis, those who develop infection in the ICU are under close supervision through iterative clinical assessment and monitoring of various blood markers. The onset of infection should therefore be identified more easily and more quickly in this setting. However, usual infection-related signs and symptoms can be missing in such patients because of the deep alterations of their immune status as well as the exposure to specific therapies and procedures. As a result, the management of nosocomial infection is probably delayed in number of cases. On the other hand, the overuse of antibiotics is a common feature in ICU [5]. This results from the lack of specificity of the clinical diagnosis of infection, and the fear of not treating life-threatening infection in critically ill patients.

Efforts have been made to develop new biomarkers that accurately predict sepsis occurrence in such patients. Among them, serum procalcitonin (PCT) is one of the most promising [6]. One recently published study showed that daily monitoring of PCT could allow medical staff to identify patients with the highest risk of mortality [7]. In addition, several reports have shown that both PCT elevation and time course could be helpful in differentiating between patients who acquired infection in the ICU and those who did not [8-11]. However, most of these studies included post-operative patients, and only very few of them provided the data necessary to assess PCT accuracy in discriminating suspected from proven sepsis. We therefore addressed this issue in an observational cohort study conducted in our medical ICU.

## Methods

The study was conducted from January, 2006 to May, 2007 in a 15-bed medical ICU in a teaching hospital.

### Patients

Procalcitonin is routinely assessed in every patient with a clinically suspected infection [12]. In addition, PCT daily measurement is used, in addition to clinical judgment, to assess prognosis, to predict unfavorable outcome and to customize the length of antibiotic therapy if necessary, as previously published [7].

Every consecutive patient with a clinical suspicion of VAP as defined below was prospectively enrolled in an observational study that aimed to evaluate the effects of implementing local guidelines for the diagnosis and the management of VAP. Notably, PCT measurement was performed daily from the day of the clinical suspicion in all of these patients as a part of the study protocol. This study was approved by the Local Ethic Committee (Comité de Protection des Personnes, C.H.U. Dijon). No informed consent was required. Patients in whom the diagnosis of VAP was considered unlikely as detailed below and regardless of the PCT level, in the absence of another suspected or proven infection source, formed a control group of patients with unconfirmed infection.

Over the same period, the clinical and biological characteristics of patients with a clinical suspicion of infection related to bacteremia, as defined below, were prospectively recorded as part of a regular surveillance study about blood stream infection.

Only patients with nosocomial bacteremia (see definition below) were considered for being included in the study.

Among the eligible patients, only those with one PCT measurement obtained the day the infection was suspected and at least 2 measurements taken within a 3-day period preceding the event were kept for further analysis. In addition, patients with candidemia, a recent history (i.e., within the past 7 days) of proven infection, resuscitated cardiac arrest or abdominal surgery were excluded.

### Definitions

Ventilator associated pneumonia was considered in every patient submitted to mechanical ventilation for more than 2 days if the following conditions were met: (i) new lung infiltrate on the chest X-ray; (ii) positive tracheal aspirate cultures (> 10^6 ^CFU/mL); (iii) modified Clinical Pulmonary Infection Score (CPIS) ≥ 6 points (i.e., a CPIS [13] in which tracheal aspirates culture was considered as positive if at least 10^6 ^CFU/mL bacteria were recovered); (iv) at least 2 S.I.R.S. criteria.

One episode of bacteremia was defined as the recovery of any bacterial species, in one or more blood cultures. Patients in whom *Staphylococcus *non-*aureus *were isolated in blood cultures were not eligible, except if at least 2 consecutive samples grew for the same species harboring the same antibiotic resistance pattern. Blood samples were obtained by venous puncture before being processed using the BACTEC system based both on standard aerobic and anaerobic media coupled with the 9240 automate (Beckton Dickinson Diagnostic Instrument System, Paramus, NJ, USA). Bacteria identification was based on standard methods. The onset of bacteremia was defined as the day when the first positive blood culture was obtained. Bacteremia was considered as nosocomial if the onset occurred at least 48 hours after ICU-admission.

### Measurements of PCT level

The Kryptor^® ^immunoassay was used according to the manufacturer's instructions (Brahms, Hennigsdorf, Germany). The functional sensitivity of the assay is 0.06 ng/mL. Patients were excluded from further analysis if the PCT measurement was not performed within the 12 hours following the blood sampling because of the risk of a false-negative result.

### Statistical analysis

Values are expressed as mean ± SD unless otherwise stated. Patients with nosocomial infection were compared to those without from D-3 to D0. For patients in whom PCT was obtained on D-1, the ΔPCT_D-1, D0 _(i.e., PCT_D0 _- PCT_D-1_) was calculated. The same calculation was done for the patients with PCT measurement available on D-3 and/or D-2, so that ΔPCT_D-2, D0 _and ΔPCT_D-3, D0 _were obtained. Continuous variables were compared with the Mann Whitney U test. Categorical variables were compared using the Chi2-test. In addition, the conformity with the linear gradient of each continuous variable was checked. If the linear model was not appropriate to describe its variations, the variable was transformed according to the parsimonious rule. As a result, the log_10_PCT was considered instead of the PCT. The candidate variables were then manually entered into a logistical regression model if the associated regression coefficient had a *p *value less than 0.20 by univariate analysis, and then removed if a *p *value less than 0.05 was obtained by multivariate analysis.

The diagnosis accuracy of serum PCT for the diagnosis of nosocomial infection was expressed as the area under the corresponding receiver operating characteristic curve (AUROCC). The optimal threshold value was then selected. Sensibility, specificity, positive predictive value, negative predictive value and likelihood ratios were then calculated. The diagnosis accuracy of each other relevant marker of infection was compared to those achieved by PCT through the corresponding AUROCC comparisons.

A *p *value < 0.05 was considered as statistically significant for all analyses. STATA software was used for all analyses (STATA Statistical Package, College Station, Tex., USA).

## Results

### Patients

**Figure 1:**
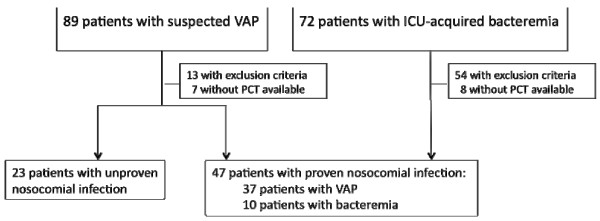
**Flow chart of the study**. PCT: procalcitonin; VAP: Ventilator Associated Pneumonia; ICU: Intensive Care Unit.

Over the study period, VAP was clinically suspected in 89 patients. According to the aforementioned criteria, VAP was confirmed in 57 patients. Among them, 13 could not be included because of the presence of at least one of the exclusion criteria. In addition, PCT was not detected in another 7. As a result, 37 patients with VAP were included in the proven nosocomial infection group. During the same period, 123 episodes of bacteremia were recorded, among which 72 were considered ICU-acquired. Among these eligible patients, only 10 could be kept for further analysis. Actually, 54 met exclusion criteria and the required PCT measurements were not available in the 8 remaining ones (Figure 1).

Twenty-three of the 32 patients with unconfirmed VAP were considered for inclusion in the group of patients with suspected but unproven nosocomial infection. The remaining ones were excluded in accordance with the aforementioned criteria.

As shown in Table 1, the baseline characteristics of patients with proven infection were no different from those without, except in terms of gender.

**Table 1:** Baseline characteristics and outcome of the included patients.

Mean (SD) or number (%)	Nosocomial infection *n *= 47	Unproven nosocomial infection *n *= 23	*p*
Age (year-old)	65.4 (16.2)	58.8 (16.1)	0.113
Female/Male	10 (21.3)/37 (78.7)	11 (47.8)/12 (52.2)	0.046
SAPS II	48.7 (14.6)	50.9 (16.1)	0.570
Underlying condition			
Cardiovascular disease	16 (34.0)	7 (30.4)	0.975
Respiratory disease	12 (25.5)	9 (39.1)	0.374
Cirrhosis	5 (10.6)	1 (4.3)	0.668
Diabete mellitus	7 (14.9)	2 (8.7)	0.728
Malignancy	2 (4.2)	4 (17.4)	0.165
Chronic renal failure	4 (8.5)	0 (0.0)	0.372
Admission diagnosis			
Sepsis	17 (36.2)	10 (43.5)	0.742
Respiratory distress	20 (42.5)	9 (39.1)	0.988
Shock	17 (36.2)	7 (30.4)	0.836
Neurological disorder	14 (29.8)	7 (30.4)	0.999
Acute renal failure	12 (25.5)	3 (13.1)	0.376
Post-operative	3 (6.4)	1 (4.3)	0.999
ICU length of stay (N. of days)	31.0 (16.2)	30.3 (15.6)	0.861
ICU mortality	42.5%	21.7%	0.150

SAPS: Simplified Acute Physiologic Score; ICU: Intensive Care Unit.

At the time nosocomial infection was suspected, the length of ICU stay in the two groups was comparable (Table 2). However, while no difference was found regarding disease severity as assessed using the SOFA score, there was a trend towards lower arterial blood pressure, a greater concentration of blood lactates and a significantly lower platelet count in the confirmed infection group. It is worth noting that within the proven infection group, patients with either VAP or bacteremia were similar, except a trend toward a greater PaO_2_/FIO_2 _ratio in the latter (Table 3).

**Table 2:** Main characteristics of the included patients at the time nosocomial infection was clinically suspected.

Mean (SD) or number (%)	Nosocomial infection *n *= 47	Unproven nosocomial infection *n *= 23	*p*
Time elapsed from ICU admission (N. of days)	16.1 (10.8)	13.5 (7.9)	0.307
Mechanical ventilation length (N. of days)	18.2 (16.5)	13.5 (9.0)	0.214
Temperature (°C)	37.6 (1.5)	37.6 (1.3)	0.958
Heart rate (bpm)	110 (23)	105 (25)	0.387
Respiratory rate (bpm)	30 (8)	28.1 (7)	0.367
PaO_2_/FIO_2 _(mmHg)	226 (125)	217 (119)	0.777
MAP (mmHg)	64 (15)	73 (20)	0.061
Vasopressor requirement (Yes/No)	20 (42)	6 (26.1)	0.282
SOFA score	6.9 (4.1)	7.2 (3.9)	0.706
Previous exposure to steroids	20 (42)	6 (26.1)	0.282
WBC count (10^3 ^cells/mm^3^)	17.1 (11.8)	13.6 (7.0)	0.226
CRP (mg/L)	130.1 (104.3)	95.6 (60.5)	0.238
PCT D0 (ng/mL)	5.5 (9.4)	0.7 (1.2)	0.018
ΔPCT_D-1, D0 _*	+5.8 (1.3)	-0.5 (10.4)	0.035
ΔPCT_D-2, D0 _**	+2.7 (6.9)	-0.9 (2.2)	0.048
ΔPCT_D-3, D0 _***	+4.3 (10.1)	-1.2 (2.7)	0.032
Platelet count (cell/mm^3^)	185,459 (118,375)	235,935 (183,485)	0.028
Creatininemia (μmol/L)	172.6 (136.9)	146.3 (164.9)	0.492
Lactate (mmol/L)	2.1 (0.9)	1.5 (0.4)	0.055

ICU: Intensive Care Unit; MAP: Mean Arterial Pressure; SOFA: Sepsis-related Organ Failure Assessment; WBC: White Blood Cell; CRP: C-reactive Protein; PCT: Procalcitonin; ΔPCT_D-1, D0 _= PCT D0 – PCT D-1; D0: day when infection is clinically suspected.

*available in 45 out of the 70 included patients; **available in 44 out of the 70 included patients; ***available in 51 out of the 70 included patients.

**Table 3:** Main characteristics of the included patients with either proven VAP or bacteremia at the time it was clinically suspected.

Mean (SD) or number (%)	VAP *n *= 37	bacteremia *n *= 10	*p*
Time elapsed from ICU admission (N. of days)	16.8 (11.1)	13.5 (9.8)	0.391
Temperature (°C)	37.4 (1.6)	38.2 (1.3)	0.163
Heart rate (bpm)	110 (23)	112 (23)	0.828
Respiratory rate (bpm)	30 (9)	31 (3)	0.652
PaO_2_/FIO_2 _(mmHg)	208 (110)	292 (157)	0.074
MAP (mmHg)	63 (15)	69 (14)	0.241
Vasopressor requirement (Yes/No)	20 (42)	6 (26.1)	0.282
SOFA score	7.0 (4.6)	5.9 (4.7)	0.489
Previous exposure to steroids	20 (42)	6 (26.1)	0.282
WBC count (10^3 ^cells/mm^3^)	17.9 (12.8)	14.3 (7.3)	0.394
CRP (mg/L)	137.6 (111.1)	98.6 (66.8)	0.382
PCT D0 (ng/mL)	4.7 (8.4)	8.5 (12.3)	0.261
ΔPCT_D-1, D0 _*	+4.7 (9.5)	+10.9 (13.5)	0.189
Platelet count (cell/mm^3^)	256,912 (207,429)	262,800 (231,402)	0.939
Creatininemia (μmol/L)	166.8 (131.6)	192.8 (160.2)	0.602
Lactate (mmol/L)	2.0 (0.9)	2.6 (1.1)	0.258

**Figure 2:**
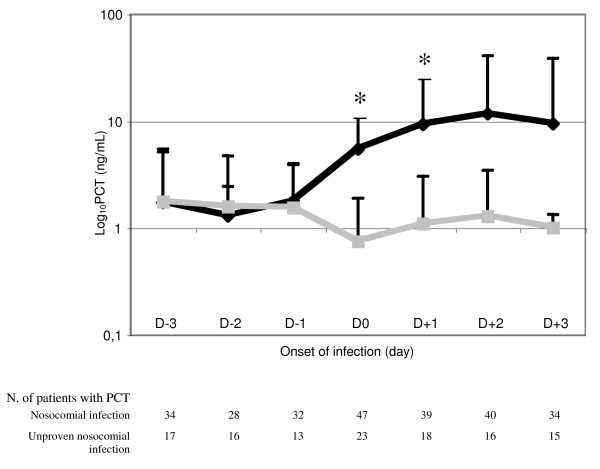
**Kinetic of procalcitonin in the serum of patients with (black line) or without (gray line) nosocomial infection in the ICU**. PCT: procalcitonin.*: *p *< 0.05.

Even though patients from both groups were found to be comparable when considering SIRS criteria, PCT on the day infection was suspected was significantly higher in patients with proven infection than in those without (5.5 [9.4] vs. 0.7 [1.2] ng/mL; *p *= 0.018) (Figure 2). We were also able to assess PCT variation during the 24 hours preceding the clinical suspicion of infection in 45 out of the 70 patients. It is worth noting that the absolute difference between D0 and D-1 was found to be significantly greater in the population with proven infection than in those without (+5.8 [1.3] vs. -0.5 [10.1]; *p *= 0.035). Interestingly, we found that PCT variation between D0 and D-2, as well as between D0 and D-3, were also markedly different between these 2 groups of patients (Table 2).

In an attempt to remove any potential confounding variable, a multivariate analysis model was then constructed as detailed in the methods section. An important finding was that PCT on day 0 was the only independent risk factor associated with proven infection (Odd ratio = 7.69, 95% CI: 2.50–25.0; *p *< 0.001).

**Figure 3:**
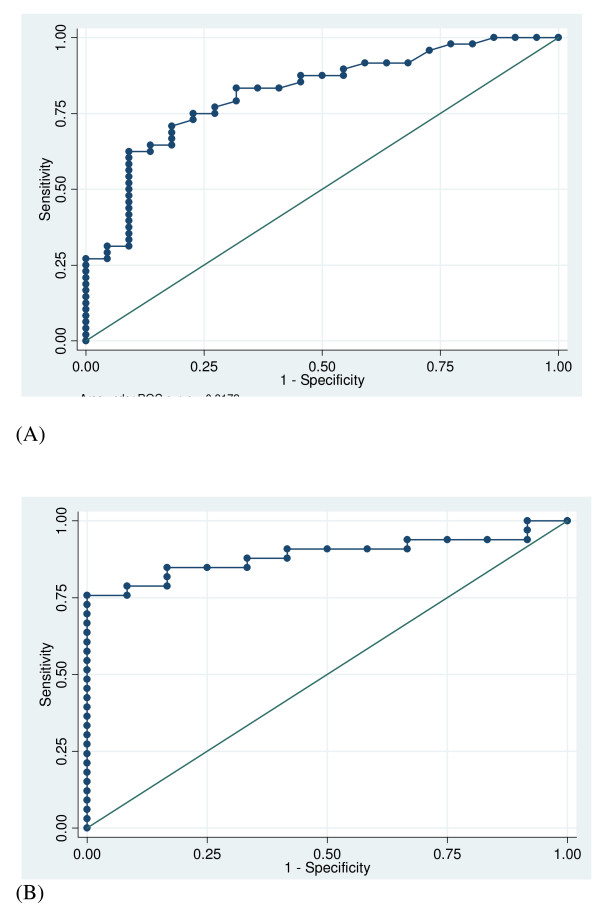
ROC curves of PCT D0 (A) and ΔPCT_D-1, D0 _*(B) for differentiating between patients with or without nosocomial infection in the ICU: AUROCC = 0.80; 95% CI, 0.68–0.91 and 0.89; 95% CI, 0.79–0.98, respectively. ICU: intensive care unit; PCT: procalcitonin; ΔPCT_D-1, D0 _= PCT D0 – PCT D-1; D0: day when infection is suspected. *available in 45 out of the 70 included patients.

Then, the diagnostic value of PCT was evaluated through the construction of the corresponding ROC curves. At D0, the area under the ROC curve was 0.80 (95% CI, 0.68–0.91). A cut-off value of 0.44 ng/mL provides sensitivity and specificity of 65.2%, 83.0%, respectively (Table 4). Although it could be calculated in only 45 patients, ΔPCT_D-1, D0 _(i.e., PCT variation between D-1 and D0) was also found to be accurate in differentiating between proven and refuted nosocomial infection (Figure 3). Thus, the corresponding AUROCC was 0.89 (95% CI, 0.79–0.98), and positive and negative predictive values reached 100% and 68%, respectively, if the +0.26 ng/mL threshold value was used. The results obtained while considering ΔPCT_D-2, D0 _and ΔPCT_D-3, D0 _are presented in Table 4. Finally, when compared with body temperature, leukocyte count and CRP, PCT was the most accurate marker of infection. It is worth noting that the diagnostic value of these markers as assessed through the corresponding ROC curve was poor. The AUROCC were 0.54 (0.40–0.68), 0.59 (0.44–0.75) and 0.58 (0.42–0.75) for temperature, leukocytes and CRP, respectively (*p *< 0.01 for all as compared with the AUROCC of PCT on D0).

**Table 4:** Diagnostic accuracy of serum PCT for the diagnosis of nosocomial infection.

PCT value	AUROCC (95% CI)	cut-off value	Sensitivity (%)	Specificity (%)	Positive predictive value (%)	Negative predictive value (%)	LR+	LR-
PCT D0	0.80 (0.68–091)	0.44	65.2	83.0	83.0	65.2	3.83	0.42
ΔPCT_D-1, D0 _*	0.89 (0.79–0.98)	+0.26	75.8	100	100	68.0	NA	0.24
ΔPCT_D-2, D0 _**	0.89 (0.75–0.96)	+0.20	67.9	100	100	64.0	NA	0.32
ΔPCT_D-3, D0 _***	0.84 (0.71–0.93)	+0.21	61.8	100	100	56.7	NA	0.38

PCT: procalcitonin; AUROCC: Area Under the Receiver Operating Characteristic Curve; CI: Confidence Interval; LR: Likelihood Ratio; NA: not applicable

ΔPCT_D-1, D0 _= PCT D0 – PCT D-1; D0: day when infection is clinically suspected.

*available in 45 out of the 70 included patients; **available in 44 out of the 70 included patients; ***available in 51 out of the 70 included patients.

It is worth noting that similar findings were obtained when the sole patients with VAP were considered (data not shown).

## Discussion

We show herein that PCT could be helpful for the early detection of septic complications in critically ill medical patients. Thus, the level of PCT obtained the day the infection is suspected is a better predictor than are clinical parameters such as body temperature and other elements of the SIRS. Our findings also suggest that low cut-off values could be used for the diagnosis of ICU-acquired infection in this particular setting. This illustrates the difficulty of determining PCT cut-off values for the diagnosis of ICU-acquired infection in critically ill patients.

The kinetic analysis of PCT might help to circumvent this drawback and should be preferred in this setting. Indeed, we showed here that a PCT elevation of at least 0.26 ng/mL over the previous 24 hours was strongly associated with the diagnosis of infection since the positive predictive value reached 100%. It is, however, worth noting that the negative predictive value of PCT using such threshold values was quite low. This reflects the low sensitivity of PCT in our study population and thus underlines the risk of false negative results.

Our results agree in part with those reported by Luyt *et al *[14]. These authors found that the positive predictive value of an increase in PCT within the previous 5 days reached 79% in 73 patients with clinically suspected VAP. In contrast, the diagnosis accuracy of PCT elevation on the day VAP was clinically suspected was poor. Thus, positive and negative predictive values were 43% and 53%, respectively, if a threshold of 0.5 ng/mL was applied. The study population was, however, markedly different from ours since it comprised patients with sepsis on admission. In addition, half of them had undergone surgery prior to VAP. We believe that these factors could have led to an underestimation of the diagnostic value of PCT, and could account for the discrepancies between these findings and ours [15,16]. Other authors have reported that the combination of PCT and CPIS could provide very high predictive values regarding the diagnosis of VAP, but in these studies more than half of the eligible patients were excluded because of previous sepsis [17].

It is well established that such conditions (i.e., prior surgery, sepsis, renal dysfunction, etc...) could diminish the diagnostic value of PCT. Thus, greater values should be expected in post-operative patients as well as in patients with acute renal failure [10,11,15]. We have also shown that the diagnosis accuracy of PCT in critically ill patients with bacteremia was lower if there had been a previous episode of sepsis and could be different according to the isolated pathogen [18,19]. The assessment of PCT kinetics could therefore be proposed to overcome this drawback. Although it could not be determined in all our patients, PCT elevation within the 24 hours preceding the clinical suspicion of infection seems to be strongly associated with the risk of proven infection.

Several limitations should however be mentioned. First, our findings were obtained from a single center in a selected population and cannot necessarily be extended to other critically ill patients. Accordingly, the study design led to the exclusion of numbers of early-onset ICU-acquired infection episodes. As suggested by the long time generally elapsed between admission and clinical suspicion of infection, our findings might be applicable to the only patients with late-onset nosocomial infection. Second, PCT on D-1 could not be obtained in all of the included patients, which could have altered our findings. Third, most of the proven infections were VAP. One could therefore argue that we did not use a reliable diagnostic tool since no invasive procedure had been conducted. Thus, recently published studies have emphasized the lack of diagnostic value of the CPIS [20,21]. As a result, episodes of VAP might have been missed in the group of unconfirmed infection while an incorrect diagnosis of proven infection may have been made in the remaining patients. We should admit therefore that our findings regarding PCT value might have been different if VAP diagnosis had relied on other criteria. In addition, we did not consider nosocomial infections caused by virus, given the expected lack of reliability of PCT as a diagnostic tool in this context. Finally, since PCT elevation is thought to be greater in the patients with bacteremia than in those with VAP, one cannot exclude that PCT levels had been overestimated in the proven infection group. However, similar findings were obtained when the only patients with either suspected or proven VAP were considered. Finally, it is worth noting that PCT daily measurement cost could compromise the translation of our findings into the clinical practice. However, our findings suggest that PCT measurement twice a week instead of once a day could also provide relevant information regarding kinetic at lower cost.

## Conclusion

Our results suggest that even when mild, any increase in PCT in a critically ill patient should contribute to warn the physician of the risk of infection, in addition to clinical findings, after excluding other obvious causes of PCT elevation such as recent surgery or cardiac arrest. Additional larger studies are needed to confirm these findings and to establish the basis of an interventional study that would aim to compare the management of critically ill patients with or without the information brought by daily monitoring of PCT levels.

## Competing interests

PEC has received payments from Brahms (Hennigsdorf, Germany) to attend one meeting about sepsis management. The other authors have not received any payment.

## Authors' contributions

PEC designed the study, analyzed the data and drafted the manuscript. EK collected the data and participated to their interpretation. SA performed the statistical analysis. JPQ, JMD, SP and BB participated to the redaction of the manuscript. NOO managed the activity of the Immunology Laboratory. All authors read and approved the final manuscript.

## Pre-publication history

The pre-publication history for this paper can be accessed here:

http://www.biomedcentral.com/1471-2334/9/49/prepub
